# Surface Plasmon Resonance Analysis for Evaluating ASO Targeting Structured RNA

**DOI:** 10.3390/mps9020048

**Published:** 2026-03-15

**Authors:** Tomohiro Shinozaki, Takuya Hasegawa, MST Tahmina Akter, Kazuyuki Kumagai, Youichi Suzuki, Taiichi Sakamoto

**Affiliations:** 1 Department of Life Science, Faculty of Advanced Engineering, Chiba Institute of Technology, 2-17-1 Tsudanuma, Narashino 275-0016, Chiba, Japan; s20c2066tm@chibatech.ac.jp (T.S.); s17c2094ap@chibatech.ac.jp (T.H.); tahmina.akter_mst@it-chiba.ac.jp (M.T.A.); k-kumagai@musashino-u.ac.jp (K.K.); 2 Laboratory of Biosafety Research, Faculty of Medicine, Osaka Medical and Pharmaceutical University, 2-7 Daigaku-machi, Takatsuki 569-8686, Osaka, Japan; youichi.suzuki@ompu.ac.jp

**Keywords:** antisense oligonucleotide (ASO), surface plasmon resonance (SPR), structured RNA

## Abstract

Antisense oligonucleotides (ASOs) are nucleic acid therapeutics that regulate gene expression through sequence-specific hybridization with target RNA. Under physiological conditions, many target RNAs adopt higher-order structures, which can strongly influence ASO accessibility and binding behavior. Although UV melting analysis is widely used to evaluate the thermal stability of ASO/RNA duplexes, this approach does not adequately account for the structural features of target RNAs. In this study, we investigated the utility of surface plasmon resonance (SPR) analysis as an in vitro method to evaluate ASO binding while considering RNA structural constraints. Multiple ASOs were designed to target PRF84, an 84-nucleotide RNA motif that induces −1 programmed ribosomal frameshifting in HIV-1 *gag*-*pol* expression. SPR analyses were performed to compare ASO interactions with complementary RNA fragments and with structurally folded PRF84. The results demonstrated that identical ASOs exhibited distinct binding behaviors depending on whether the target was a complementary RNA or PRF84, indicating that RNA structure significantly affects ASO binding. These findings suggest that SPR analysis enables the evaluation of ASO–RNA interactions taking structure into account, and may be a useful alternative approach to conventional UV melting analysis-based ASO screening.

## 1. Introduction

Antisense oligonucleotides (ASOs) are a class of nucleic acid therapeutics that regulate gene expression through sequence-specific binding to target mRNAs via Watson–Crick base pairing. In recent years, several ASO therapeutics, such as Mipomersen and Inotersen, have been approved, establishing the clinical application of ASOs [1,2]. Approved ASOs commonly incorporate chemical modifications such as phosphorothioate (PS) backbone modifications and locked nucleic acid (LNA) sugar modifications, which contribute to improved nuclease resistance, pharmacokinetic properties, and binding affinity to target RNA [3,4]. The binding affinity and specificity of ASOs strongly depend on sequence design, and the selection of ASOs with high affinity to the target RNA is a critical determinant of their therapeutic efficacy. However, designed ASOs do not always exhibit the expected activity, which is thought to be partly due to the structure of the target RNA. Therefore, improved screening strategies are needed.

In the initial screening of ASO sequence design, the melting temperature (*T*_m_) obtained by UV melting analysis is widely used to evaluate the thermal stability of duplexes between ASOs and complementary RNA. However, under physiological conditions, target RNAs frequently form higher-order structures such as stem–loops or pseudoknots, and these structures can reduce the accessibility of ASOs to their target RNAs and thereby reduce their gene silencing activity [5,6,7,8]. Consequently, *T*_m_ analysis of the duplexes between ASOs and complementary RNA cannot sufficiently capture the binding behavior of ASOs to structured target RNAs. Therefore, it is necessary to establish ASO evaluation methods that take the structural features of target RNA into account.

Surface plasmon resonance (SPR) was first reported for biosensing in 1983 and has since developed as a method for analyzing biomolecular interactions [9,10,11], and in recent years has also been used for imaging [12,13,14]. Although various methods for analyzing biomolecular interactions have been reported [15,16], SPR is one of the most commonly used methods for analyzing the biomolecular interactions due to its high throughput. It can determine binding affinity based on the dissociation constant (*K*_D_), and obtain kinetic parameters such as the association rate constant (*k*_on_) and dissociation rate constant (*k*_off_). SPR has been applied to analyze protein–protein interactions [17,18], nucleic acid–protein interactions [19,20,21], and interactions between small molecules and proteins [22,23]. It has also been applied to analyze nucleic acid–nucleic acid interactions. Palau et al. used SPR to show that the stem–loops of HCV genomic RNA hybridize to each other through loop–loop interactions [24]. Dausse et al. performed SPR-based SELEX to select RNA aptamers against a structured RNA derived from *XBP1* pre-mRNA [25]. Based on these studies, we decided to use SPR for ASO screening and evaluation. Recently, Stulz et al. reported a biophysical screening strategy using differential scanning fluorimetry, circular dichroism, isothermal titration calorimetry (ITC), small-angle X-ray scattering, and SPR to examine the affinity of ASOs for their target RNA [26]. In the paper, they analyzed the interaction between ASOs and target RNAs using multiple techniques, focusing on differences in chemical modifications, but not on the structure of the target RNA.

In this study, we focused on SPR analysis as a method to evaluate the binding behavior of ASOs toward structured target RNA. As a model sequence forming a stem–loop structure, we selected an RNA motif that induces −1 programmed ribosomal frameshifting (PRF) in the HIV-1 *gag*-*pol* region [27,28,29]. We designed multiple ASOs against this target, analyzed their interactions by SPR, and compared them with the interactions observed with short fragments of complementary RNA. Through this approach, we demonstrate the utility of SPR analysis as an in vitro method for evaluating ASO binding behavior, taking the structural features of target RNA into account.

## 2. Materials and Methods

### 2.1. ASOs and Target RNA

**Figure 1:**
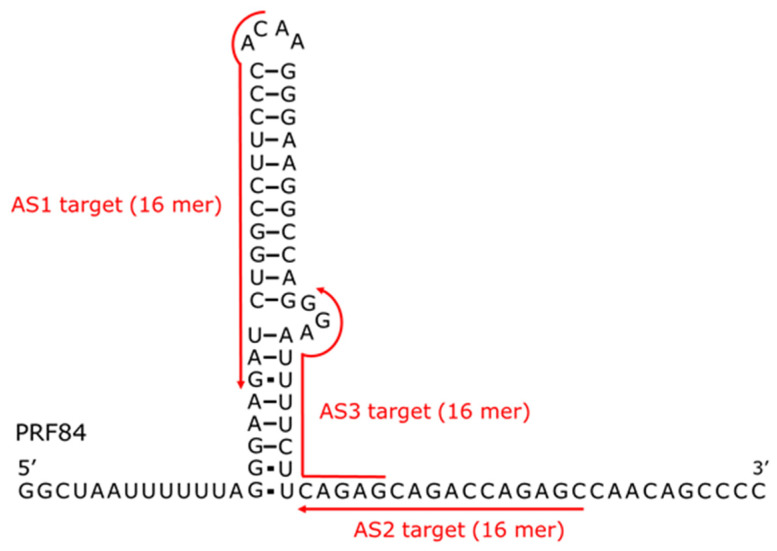
Predicted secondary structure of the target RNA PRF84 and design of ASOs. Target sequence of the ASOs is indicated by red.

As a model for the interactions between ASOs and structured RNA, we selected the HIV-1 *gag*-*pol* region RNA that induces PRF and three complementary ASOs. An 84-nucleotide RNA (PRF84) and ASOs (AS1, AS2, AS3) complementary to different regions of PRF84 were designed (Figure 1 and Table 1). Gapmer-type ASOs (AS1Gap, AS2Gap, and AS3Gap) were further designed by introducing PS backbone modifications and LNA modifications at the three terminal nucleotides at both ends. Furthermore, short RNA fragments (RNA1, RNA2, RNA3) complementary to the ASOs were also designed. For immobilization on the SPR sensor chip SA, the complementary RNAs and PRF84 were biotinylated at the 3′ end. All oligonucleotides were purchased from Hokkaido System science Co., Ltd. (Sapporo, Japan).

### 2.2. UV Melting Analysis

ASOs and non-biotinylated RNA fragments were dissolved in 1 × PBS [137 mM NaCl, 2.7 mM KCl, 10 mM Na_2_HPO_4_, 1.8 mM KH_2_PO_4_ (pH 7.4)] and degassed for 30 min using an evaporator. To form double-stranded ASO/RNA, 2 μM of each of ASO and RNA were incubated at 95 °C for 5 min, and then slowly cooled to room temperature. Using V-730BIO (JASCO Co., Tokyo, Japan) with an eight-sample cell changer PAC-743 (JASCO Co., Tokyo, Japan) with quartz of 1 cm pathlength, absorbance at 260 nm was measured as a function of temperature from 15 °C to 95 °C (temperature increase rate = 0.5 °C/min). The *T*_m_ value was estimated from the second derivative of melting curve.

### 2.3. SPR Analysis

SPR measurements were performed at 25 °C using BiacoreX100 (Cytiva, Marlborough, MA, USA). The 3′-biotinylated RNA fragments were immobilized on the surface of a sensor chip SA (Cytiva, Marlborough, MA, USA) to Flow cell 2 as a ligand at a level of approximately 300–600 Resonance units (RUs). The 3′-biotinylated PRF84 was immobilized on the surface of a sensor chip SA to Flow cell 2 as a ligand at a level of approximately 1500 RU. To compensate for the expected lower binding affinity of ASOs to structured RNA compared with complementary RNA fragments, a higher immobilization level was used for PRF84. After immobilization, the sensor chip was washed and equilibrated with SPR buffer (1 × PBS). Subsequent analyses were performed using a single-cycle kinetics method at 40 μL/min. The association time was 120 s, and the dissociation time was 1800 s. ASOs in SPR buffer were injected as analytes at concentrations of 87.5, 175, 350, 700, 1400 nM for RNA1, 56.25, 112.5, 225, 450, 900 nM for RNA2, 37.5, 75, 150, 300, 600 nM for RNA3, and 62.5, 125, 250, 500, 1000 nM for PRF84. The concentration range of each analyte was optimized according to the immobilization level of the RNA target and the expected binding affinity, in order to obtain reliable kinetic fitting. Analyte concentrations were increased stepwise and injected continuously without regeneration between injections. A regeneration step was performed after the final dissociation step. The sensor chip surface was regenerated with regeneration solution (50 mM NaOH) at 30 μL/min for 30 s. After regeneration, the response returned to the response before the analyte injection and plateaued, confirming baseline stability. The running buffer was run before measurements for blank. Sensorgram was analyzed with BIA evaluation software (Cytiva, Marlborough, MA, USA). A Langmuir (1:1) binding model was used to analyze the association rate constant, *k*_on_ (M^−1^s^−1^), the dissociation rate constant, *k*_off_ (s^−1^), and the dissociation constant, *K*_D_ (M). Kinetic measurements were performed in three independent measurement sessions conducted on different days using the same ligand-immobilized sensor chip. Kinetic parameters are represented by the mean ± standard error from three independent measurements.

## 3. Results

### 3.1. UV Melting Analysis of ASO/RNA Duplexes

**Figure 2:**
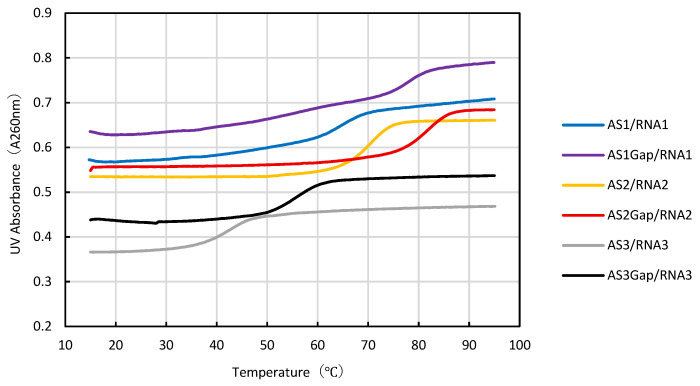
UV melting curves of ASO/RNA duplexes. Blue: AS1/RNA1. Purple: AS1Gap/RNA1. Orange: AS2/RNA2. Red: AS2Gap/RNA2. Gray: AS3/RNA3. Black: AS3Gap/RNA3.

Generally, the thermal stability of ASO/RNA duplexes has been evaluated by UV absorbance melting analysis. Therefore, we performed UV absorbance melting analysis to compare with the SPR method. Representative melting curves obtained by UV melting analysis are shown in Figure 2. The melting temperature (*T*_m_), defined as the temperature at which 50% of the duplex is denatured into single strands, reflects the thermal stability of the duplex; a higher *T*_m_ indicates greater stability.

The *T*_m_ values of the duplexes formed between unmodified ASOs and their complementary RNA fragments were 61.8 °C for AS1/RNA1, 71.7 °C for AS2/RNA2, and 43.0 °C for AS3/RNA3. This result indicates that the stability of ASO/RNA duplexes of the same length varies depending on the sequence. Furthermore, the corresponding gapmer-type ASOs exhibited higher melting temperatures, with *T*_m_ values of 73.1 °C for AS1Gap/RNA1, 81.7 °C for AS2Gap/RNA2, and 54.1 °C for AS3Gap/RNA3. Compared with the unmodified ASOs, the gapmer-type ASOs showed an increase in *T*_m_ of approximately 10 °C (Table 2). It has previously been reported that the introduction of LNAs increases *T*_m_ value [30,31].

### 3.2. SPR Analysis

#### 3.2.1. ASOs and Complementary RNA Fragments

**Figure 3:**
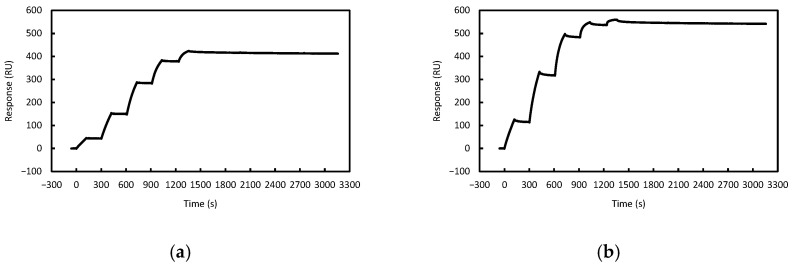

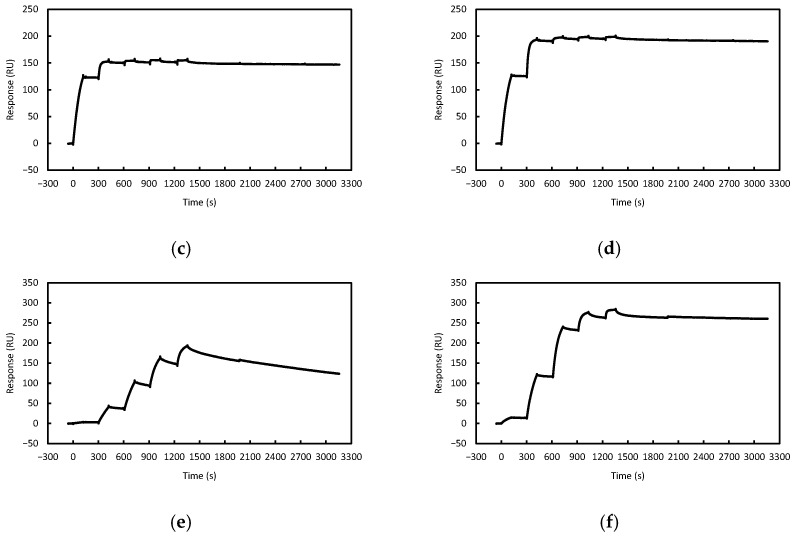
SPR sensorgrams of ASOs binding to their complementary RNA fragments. RNA fragments were immobilized on the sensor chip, and ASOs were injected at concentrations of 87.5, 175, 350, 700, 1400 nM for RNA1, 56.25, 112.5, 225, 450, 900 nM for RNA2, 37.5, 75, 150, 300, 600 nM for RNA3. Representative sensorgrams are shown. (**a**) AS1-RNA1. (**b**) AS1Gap-RNA1. (**c**) AS2-RNA2. (**d**) AS2Gap-RNA2. (**e**) AS3-RNA3. (**f**) AS3Gap-RNA3.

The interactions between each ASO and its complementary RNA fragment were analyzed by SPR. All ASOs exhibited binding responses to their corresponding complementary RNA fragments under the experimental conditions tested (Figure 3).

The dissociation constant (*K*_D_) was calculated from the ratio of the association rate constant (*k*_on_) to the dissociation rate constant (*k*_off_). For all ASOs except AS3, *k*_off_ were extremely low, approaching or below the practical detection limit of the BiacoreX100 system (*k*_off_ < 1 × 10^−5^ s^−1^). As a result, the calculated *K*_D_ values should be regarded as apparent affinities rather than precise kinetic constants (Table 3). The residual plots of the kinetic fitting are provided in Supplementary Figure S1, confirming the overall quality of the fitting.

#### 3.2.2. ASOs and PRF84

**Figure 4:**
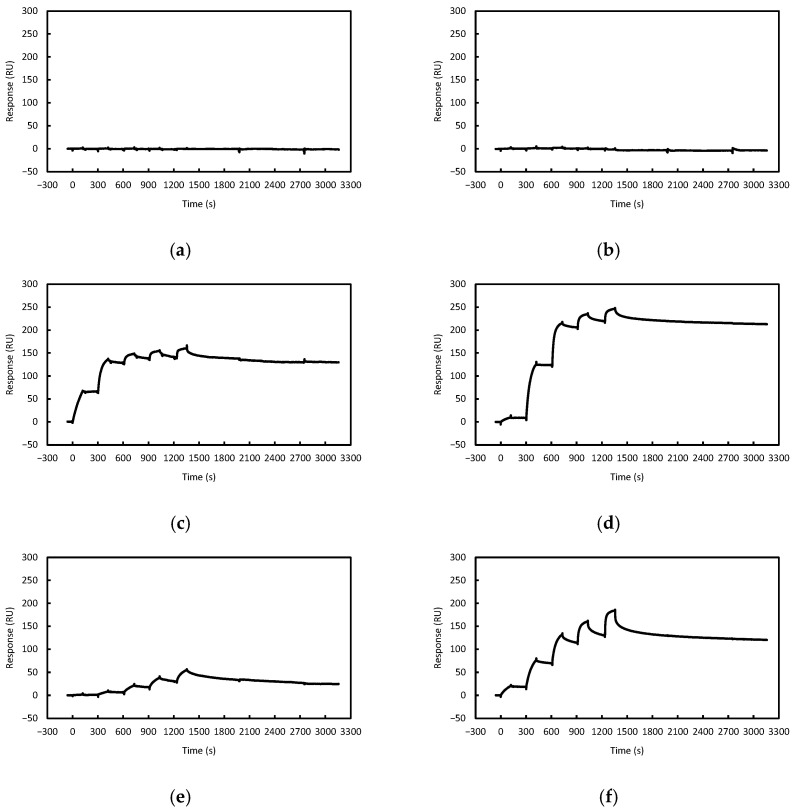
SPR sensorgrams of ASO binding to PRF84. PRF84 was immobilized on the sensor chip, and ASOs were injected at concentrations of 62.5, 125, 250, 500, and 1000 nM. Representative sensorgrams are shown. (**a**) AS1-PRF84. (**b**) AS1Gap-PRF84. (**c**) AS2-PRF84. (**d**) AS2Gap-PRF84. (**e**) AS3-PRF84. (**f**) AS3Gap-PRF84.

To evaluate ASO binding to structured RNA, PRF84 was immobilized on the sensor chip and analyzed by SPR. AS2 and AS3, as well as their corresponding gapmer-type ASOs (AS2Gap and AS3Gap), exhibited clear binding responses to PRF84. In contrast, no detectable binding was observed for AS1 or AS1Gap (Figure 4).

For AS2, AS3, AS2Gap, and AS3Gap, *K*_D_ was calculated from the ratio of *k*_on_ to *k*_off_ (Table 4).

Comparison between AS2 and AS2Gap showed that the *k*_on_, *k*_off_, and *K*_D_ values were nearly identical. However, in the SPR analysis using PRF84, the *k*_off_ values for both AS2 and AS2Gap approached the practical detection limit of the instrument and could not be reliably determined. AS3Gap exhibited an approximately 10-fold lower *K*_D_ compared with AS3. Although the *k*_on_ values were similar between AS3 and AS3Gap, the *k*_off_ value of AS3Gap was approximately 10-fold lower, indicating that the reduced dissociation rate primarily contributed to the enhanced binding affinity. The residual plots of the kinetic fitting are provided in Supplementary Figure S1.

## 4. Discussion

The gapmer-type ASOs exhibited *T*_m_ values approximately 10 °C higher than those of the corresponding unmodified ASOs, indicating that the introduction of LNA modifications at the terminal three nucleotides likely contributed to the stabilization of the ASO/RNA duplexes. These observations are consistent with previous reports describing the effects of chemical modifications on the thermal stability of ASO/RNA duplexes [30,31].

In contrast to the UV melting analysis, SPR analysis of interactions with PRF84 revealed distinct binding behaviors. Although all ASOs showed clear binding to their complementary RNA fragments, no detectable binding to PRF84 was observed for AS1 or AS1Gap. To obtain these experimental results, we investigated SPR buffer conditions. Initially, we performed SPR experiments in a solution containing 137 mM NaCl, 2.7 mM KCl, 10 mM Na_2_HPO_4_, 1.8 mM KH_2_PO_4_ (pH 7.4), 0.5 mM MgCl_2_, and 1 mM CaCl_2_, which mimics intracellular conditions. However, regenerating the SA chip did not restore the baseline of the sensorgram. Furthermore, no ASO binding to the RNA immobilized on the SA chip was observed after regeneration. This could be due to nonspecific adsorption of ASO. To address this issue, we tried using an SPR buffer containing a surfactant (0.01% Tween 20), which is commonly used in SPR analysis of proteins, but this did not resolve the issue. Next, we removed divalent metal ions from the SPR buffer, allowing us to regenerate the sensorgram and obtain highly reproducible data. The solution condition is almost identical to that used by Stulz *et al*. to analyze the interaction between ASO and complementary RNA, suggesting that an SPR buffer free of divalent metal ions is necessary for analyzing the interaction between ASO and RNA.

PRF84 is a well-characterized RNA motif that induces −1 programmed ribosomal frameshifting (PRF) during expression of the HIV-1 *gag*-*pol* gene and has been reported to adopt a secondary structure consisting of two stem regions (Figure 1) [27,28,29]. NMR analysis of the PRF stem–loop has been reported, suggesting that the upper stem is stable and its three-dimensional structure has been determined, while the lower stem is unstable [29]. In the report, the imino proton signals observed upon stem structure formation were not clearly observed in the lower stem, and our NMR analysis showed similar results (Figure S2). The broad imino proton signal that was not assigned can be assigned to the base pair in the lower stem. The broadening of the signal is thought to result from the equilibrium between the formation and dissociation states of the stem. The instability of the lower stem is thought to be due to the presence of three unstable non-Watson–Crick GU base pairs. Based on the NMR structure, the target sequence of AS1 is predicted to be largely embedded within the upper stem structure, with 14 out of 16 nucleotides forming base pairs. In contrast, the target sequence of AS2 is located entirely within a single-stranded region, while that of AS3 is partially located within the lower stem and partially within a single-stranded region. Considering the relationship between ASO target sites and the structure of PRF84, the lack of detectable binding of AS1 to PRF84 is likely attributable to reduced accessibility caused by pre-existing intramolecular base pairing at the target site. In contrast, the target sequence of AS3 contains the unstable stem and accessible single-stranded regions, which may have allowed AS3 binding to PRF84 to be detected by SPR. Consistent with this interpretation, previous studies have reported that RNA secondary and tertiary structures can significantly influence ASO accessibility, binding efficiency, and functional activity [5,6,7,8]. The binding behaviors observed in this study therefore suggest that ASO interactions with PRF84 are strongly dependent on the structural features of the target RNA. AS1Gap, like AS1, did not exhibit detectable binding to PRF84, suggesting that chemical modification alone does not necessarily overcome structural barriers to ASO accessibility in highly structured RNA targets.

Comparing the kinetic parameters of AS2 and AS2Gap binding to the complementary RNA fragment (RNA2) and PRF84 suggested that the affinity of ASOs to PRF84 was weaker than to RNA2. Considering that the target sequence of AS2 and AS2Gap is the single-stranded region of PRF84, the single-stranded region of PRF84 may form a different structure from that of the short single-stranded RNA2. However, the difference in affinity is likely due to differences in the SPR experimental conditions. The immobilization amounts of PRF84 and RNA2 were different, which resulted in different ligand densities on the sensor and may have affected the kinetic parameters. Furthermore, since PRF84 has a 10-nucleotide gap between the target sequence and the sensor chip, the difference in kinetic parameters between RNA2 and PRF84 may be caused by the flexibility and fluctuation of the RNA and the distance between the target sequence and the sensor surface. Because the SPR signal intensity is affected by the distance between the binding site and the sensor surface due to the penetration depth of the evanescent wave, the effect of the distance between the binding site and the surface of the sensor chip must be taken into account when comparing binding kinetics. The target sequences of AS1, AS2, and AS3 in PRF84 are at different distances from the sensor surface; therefore, the differences in the kinetic parameters of these ASOs may be influenced by the difference in not only structure but also distance. The significant differences in affinity for PRF84 among AS1, AS2, and AS3 are likely due primarily to differences in structure, but a more precise assessment requires extensive SPR analysis using various RNAs and ASOs.

UV thermal melting analysis is widely used as a screening method for ASO design because it can quantitatively evaluate the duplex stability between an ASO and a complementary RNA strand. However, this approach does not consider the effect of the higher-order structure formed by the target RNA under physiological conditions on ASO accessibility. This is because, when UV thermal melting is used to assess the interaction between structured RNA and ASO, it is difficult to distinguish between changes in UV absorption due to unfolding of the RNA structure and changes in UV absorption due to dissociation of the ASO. NMR may be able to distinguish between RNA unfolding and ASO dissociation, but it requires a large amount of sample, making it unsuitable for ASO screening. ITC is often compared to SPR, but ITC requires larger amounts of sample than SPR [21], making it unsuitable for ASO screening. Therefore, SPR is expected to be useful for screening ASOs that target structured RNA. Generally, the key point in protein SPR experiments is the immobilization of the protein onto the sensor chip. If the immobilization conditions are not appropriate, the protein may denature and its activity may decrease. However, a method for immobilizing biotinylated nucleic acids onto a streptavidin sensor chip is well-established and very simple. Therefore, SPR is useful for ASO screening prior to cellular experiments due to its small sample volume and high throughput.

In this study, AS1 exhibited a higher *T*_m_ with its complementary RNA fragment than AS3, yet failed to bind to PRF84, demonstrating that *T*_m_ alone is insufficient to predict ASO binding behavior toward structured RNA targets. In contrast, SPR analysis enables direct evaluation of ASO interactions with target RNAs while preserving their structural features, thereby reflecting structure-dependent accessibility. The present results demonstrate that SPR analysis can capture differences in ASO binding behavior arising from both sequence design and chemical modification in the context of structured RNA targets, highlighting its potential utility as a screening tool for structure-aware ASO design.

## Figures and Tables

**Table 1:** Sequence of oligonucleotides used in the present study.

Name	Sequence (5′→3′)
PRF84	GGCUAAUUUUUUAGGGAAGAUCUGGCCUUCCCACAAGGGAAGGCCAGGGAAUUUUCUUCAGAGCAGACCAGAGCCAACAGCCCC
RNA1	GAUCUGGCCUUCCCAC
RNA2	CAGAGCAGACCAGAGC
RNA3	GGAAUUUUCUUCAGAG
AS1	GTGGGAAGGCCAGATC
AS1Gap	g * t * g * G * G * A * A * G * G * C * C * A * G * a * t * c
AS2	GCTCTGGTCTGCTCTG
AS2Gap	g * c * t * C * T * G * G * T * C * T * G * C * T * c * t * g
AS3	CTCTGAAGAAAATTCC
AS3Gap	c * t * c * T * G * A * A * G * A * A * A * A * T * t * c * c

Lowercases letter: LNA modification; * indicates PS link.

**Table 2:** *T*_m_ values of ASO/RNA duplexes obtained from UV melting curves.

Sample	*T*_m_ (°C)
AS1/RNA1	61.8 ± 0.6
AS1Gap/RNA1	73.1 ± 0.2
AS2/RNA2	71.7 ± 1.7
AS2Gap/RNA2	81.7 ± 0.5
AS3/RNA3	43.0 ± 1.8
AS3Gap/RNA3	54.1 ± 1.6

All experiments were performed in 1 × PBS [137 mM NaCl, 2.7 mM KCl, 10 mM Na_2_HPO_4_, 1.8 mM KH_2_PO_4_ (pH 7.4)]. *T*_m_ values represent the mean ± standard error (*n* = 3).

**Table 3:** Kinetics of ASOs binding to the complementary RNA fragments.

Sample	*k*_on_ (M^−1^s^−1^) × 10^3^	*k*_off_ (s^−1^) × 10^−3^	*K*_D_ (M) × 10^−9^
AS1-RNA1	20.5 ± 1.3	0.010 ± 0.001	0.50 ± 0.02
AS1Gap-RNA1	37.8 ± 0.3	0.007 ± 0.001	0.18 ± 0.02
AS2-RNA2	467 ± 44	0.017 ± 0.001	0.038 ± 0.003
AS2Gap-RNA2	303 ± 58	0.013 ± 0.001	0.05 ± 0.01
AS3-RNA3	51 ± 6	0.26 ± 0.02	5.22 ± 0.37
AS3Gap-RNA3	165 ± 45	0.03 ± 0.003	0.19 ± 0.06

All experiments were performed in 1 × PBS [137 mM NaCl, 2.7 mM KCl, 10 mM Na_2_HPO_4_, 1.8 mM KH_2_PO_4_ (pH 7.4)], 25 °C. *k*_on_, *k*_off_ and *K*_D_ values represent the mean ± standard error (*n* = 3 independent measurement sessions).

**Table 4:** Kinetics of ASOs binding to PRF84.

Sample	*k*_on_ (M^−1^s^−1^) × 10^3^	*k*_off_ (s^−1^) × 10^−3^	*K*_D_ (M) × 10^−9^
AS2-PRF84	139 ± 17	0.06 ± 0.01	0.46 ± 0.07
AS2Gap-PRF84	135 ± 11	0.045 ± 0.001	0.34 ± 0.02
AS3-PRF84	75 ± 41	1.9 ± 0.9	29 ± 8
AS3Gap-PRF84	56 ± 18	0.14 ± 0.01	3.2 ± 1.2

All experiments were performed in 1 × PBS [137 mM NaCl, 2.7 mM KCl, 10 mM Na_2_HPO_4_, 1.8 mM KH_2_PO_4_ (pH 7.4)], 25 °C. *k*_on_, *k*_off_ and *K*_D_ values represent the mean ± standard error (*n* = 3 independent measurement sessions).

## Data Availability

All data are contained in the article and available from the corresponding authors upon request.

## Supplementary Materials

The following supporting information can be downloaded at: https://www.mdpi.com/article/10.3390/mps9020048/s1, Figure S1: Residual plots of SPR analyses; Figure S2: Imino proton spectrum of stem-loop of PRF (PRF47).

## Abbreviations

The following abbreviations are used in this manuscript:

ASO	Antisense oligonucleotide
LNA	Locked nucleic acid
PRF	Programmed ribosomal frameshifting
PS	Phosphorothioate
SA	Streptavidin
SPR	Surface plasmon resonance
*T*_m_	Melting temperature
RU	Resonance units
*K*_D_	Dissociation constant
*k*_on_	Association rate constant
*k*_off_	Dissociation rate constant
PBS	Phosphate-buffered saline
