# Supplementary material for: Surface Plasmon Resonance Analysis for Evaluating ASO Targeting Structured RNA
Source: Methods Protoc. 2026 Mar 15;9(2):48. doi: 10.3390/mps9020048 (PMC13010618; doi:10.3390/mps9020048)
Supplement: Supplementary file 1 [file mps-09-00048-s001.zip › mps-4146850-supplementary.pdf]

Supplementary Information

Surface plasmon resonance analysis for evaluating ASO targeting structured RNA

Tomohiro Shinozaki [1], Takuya Hasegawa [1], MST Tahmina Akter [1], Kazuyuki Kumagai [1], Youichi Suzuki [2], and Taiichi Sakamoto [1],*

[1]Department of Life Science, Faculty of Advanced Engineering, Chiba Institute of Technology,

2-17-1 Tsudanuma, Narashino, Chiba 275-0016, Japan

[2]Laboratory of Biosafety Research, Faculty of Medicine, Osaka Medical and Pharmaceutical University,

2-7 Daigaku-machi, Takatsuki, Osaka, 569-8686, Japan

*Correspondence: taiichi.sakamoto@chibatech.ac.jp

a AS1-RNA1

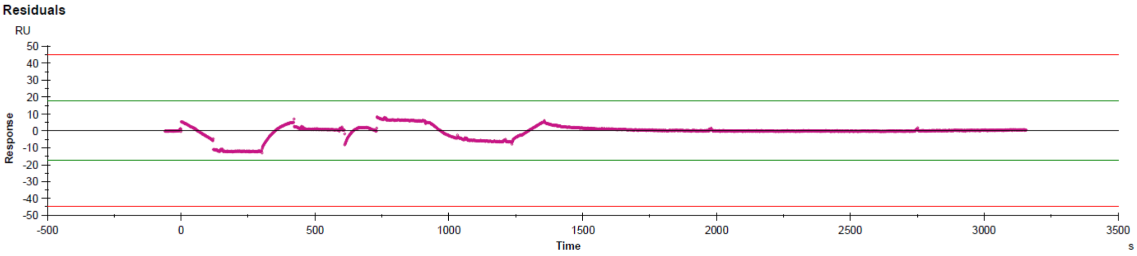

b AS1Gap-RNA1

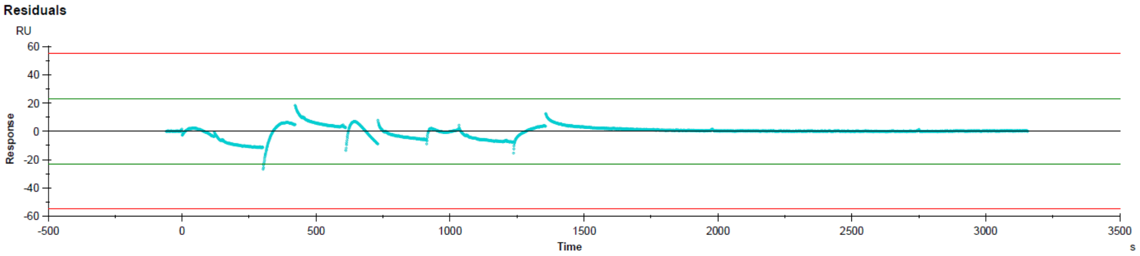

c AS2-RNA2

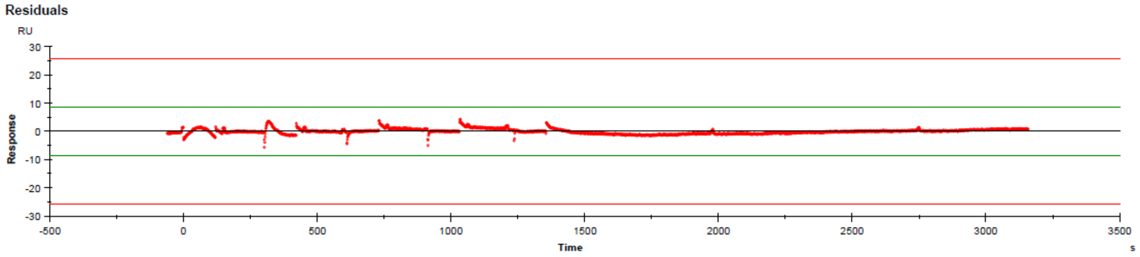

d AS2Gap-RNA2

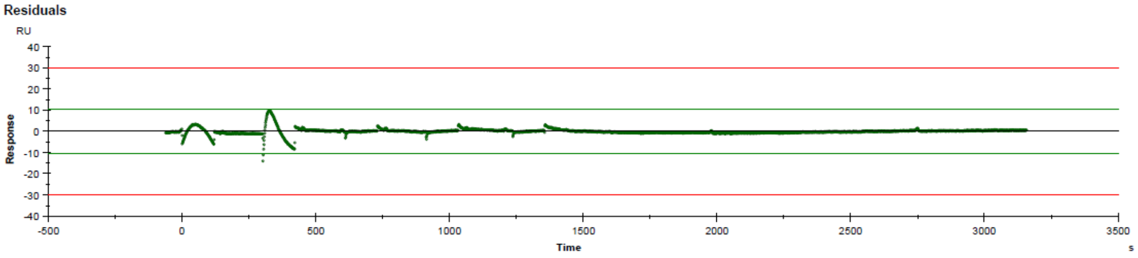

e AS3-RNA3

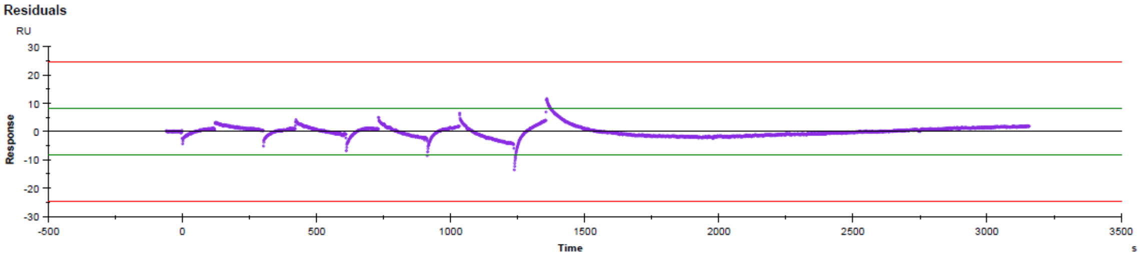

Figure S1 (continues).

f AS3Gap-RNA3

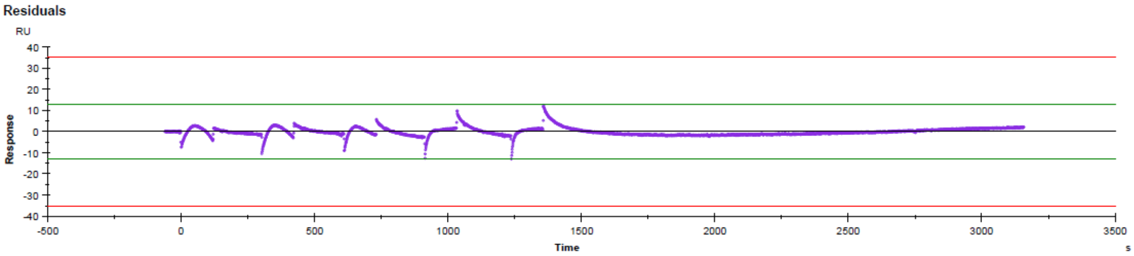

g AS2-PRF84

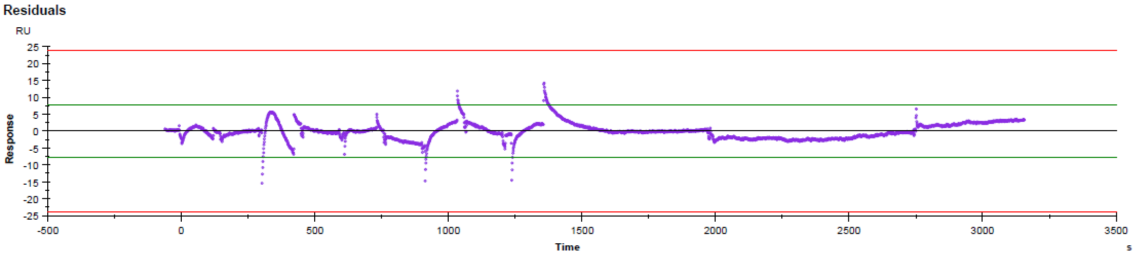

h AS2Gap-PRF84

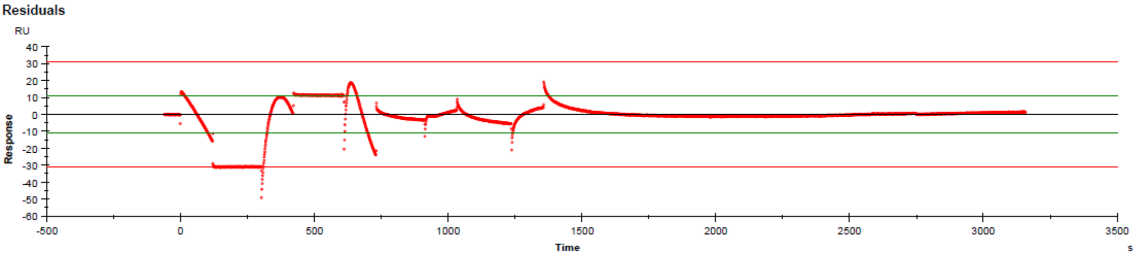

i AS3-PRF84

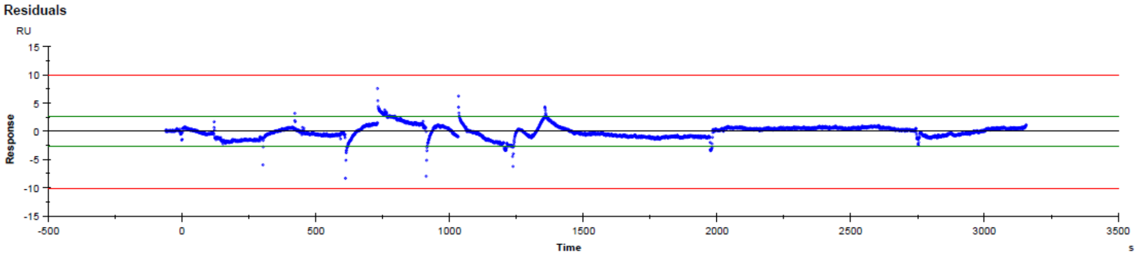

j AS3Gap-PRF84

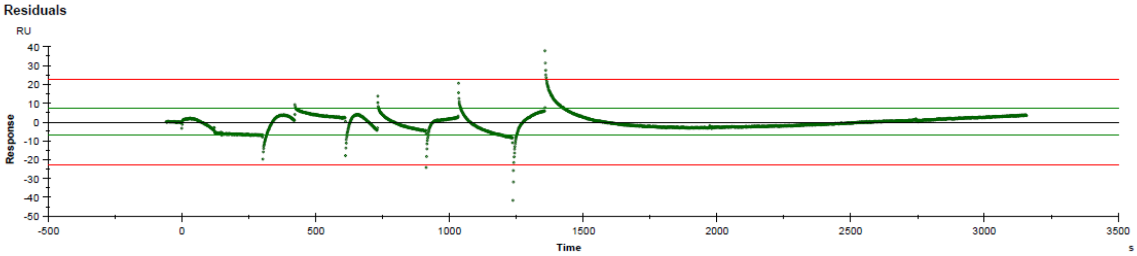

Figure S1 (continued). Residual plots of SPR analyses. (**a**)AS1-RNA1. (**b**)AS1Gap-RNA1. (**c**)AS2-RNA2. (**d**)AS2Gap-RNA2. (**e**)AS3-RNA3. (**f**)AS3Gap-RNA3. (**g**)AS2-PRF84. (**h**)AS2Gap-PRF84. (**i**)AS3-PRF84. (**j**)AS3Gap-PRF84.

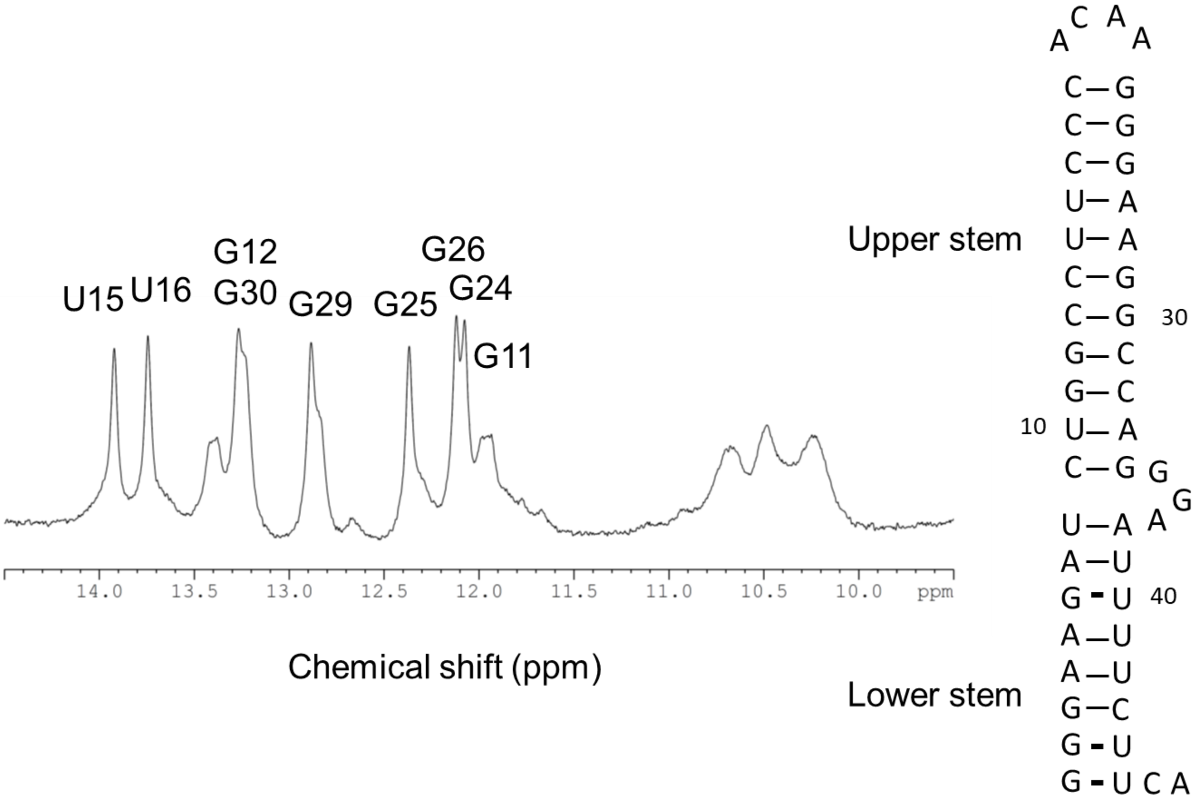

Figure S2. Imino proton spectrum of stem-loop of PRF (PRF47). PRF47 was annealed by heating at 95˚C for 5 min followed by snap-cooling on ice, and then dissolved in 20 mM sodium phosphate (pH 6.5) 50 mM NaCl. NMR spectra were measured using an AVANCE NEO 600 spectrometer (Bruker BioSpin) at a probe temperature of 10˚C using the jump-and-return schemes for water suppression. The assignments of the signals shown above the spectrum were confirmed by NOESY experiments, with reference to ref. [29].
